# Innovative Technology System to Prevent Wrong Site Surgery and Capture Near Misses: A Multi-Center Review of 487 Cases

**DOI:** 10.3389/fsurg.2020.563337

**Published:** 2020-10-23

**Authors:** David M. Gloystein, Bradley A. Heiges, David G. Schwartz, John G. DeVine, Deborah Spratt

**Affiliations:** ^1^Dwight David Eisenhower Army Medical Center, Augusta, GA, United States; ^2^Optim Orthopedics, Savannah, GA, United States; ^3^OrthoIndy, Indianapolis, IN, United States; ^4^Medical College of Georgia, Augusta University, Augusta, GA, United States; ^5^Surgical Services University of Rochester St. James Hospital, Hornell, NY, United States

**Keywords:** wrong site surgery, wrong patient, wrong side, wrong laterality, wrong procedure, near miss, patient safety, forcing function

## Abstract

**Introduction:** Wrong site surgery (WSS) is a preventable error. When these events do occur, they are often devastating to the patient, nursing staff, surgeon, and facility where the surgery was performed. Despite the implementation of protocols and checklists to reduce the occurrence of WSS, the rates are estimated to be unchanged.

**Materials and Methods:** An innovative technology was designed to prevent WSS through a systems-based approach. The StartBox Patient Safety System was utilized at six sites by 11 surgeons. The incidence of near misses and WSS was reviewed.

**Results:** The StartBox System was utilized for 487 orthopedic procedures including Spine, Sports Medicine, Hand, and Joint Replacement. There were no occurrences of WSS events. Over the course of these procedures, medical staff recorded 17 near misses utilizing the StartBox System.

**Conclusions:** StartBox successfully performed all tasks without technical errors and identified 17 near miss events. The use of this system resulted in the occurrence of zero wrong site surgeries.

## Introduction

Wrong site surgery (WSS) continues to plague medical facilities across the globe despite implementation of initiatives, checklists, and protocols. WSS refers to surgery that is incorrectly performed on the wrong side, wrong spine level, wrong anatomy, wrong patient, or the wrong procedure. Estimates on the incidence of WSS vary widely, ranging from 0.09 to 4.5 per 10,000 procedures (1–6). This potentially translates to between 683 and 34,000 wrong site surgeries per year based upon annual rates of surgical procedures in the United States. Attempting to put these wide ranges in context, Seiden suggests that WSS events occur 50 times a week or more (5); Clarke estimates that a 300-bed hospital can anticipate a report of a WSS event an average of once each (7); and Canale reports that orthopedic surgeons have a 25% chance of performing a WSS at least once in their career (8). The majority of errors are classified as wrong side, ranging from 70 to 81% of overall events (5, 7). Though small in number, the impact of WSS is large and may result in permanent injury to the patient, damaged reputations for the surgeon and surgical facility, and significantly increased medicolegal costs. When these events occur, they are often devastating to the patient, nursing staff, surgeon, and facility where the surgery was performed.

With the guidance of proper processes, checklists, and safeguards, the Joint Commission has declared that WSS is a preventable event that should never occur. In support of this expectation, the Joint Commission introduced a Universal Protocol in 2004 that provides guidelines for the fundamental elements of a WSS prevention protocol. The Universal Protocol includes requirements for marking of the surgical site, confirmation of patient identity, confirmation of the intended procedure, and review of these details among the surgical team during a time-out immediately prior to the start of surgery (9). While Universal Protocol guidelines are specific in content, implementation of the guidelines can vary widely across hospitals and surgery centers. Even when WSS prevention protocols are implemented, adherence to such protocols among staff members may not be consistent within a given facility or system (2). The Joint Commission provides causes for failures of safety protocols in the OR including distractions and rushing during time-outs (Table 1) (10). These factors may help explain the unchanging rates of WSS despite implementation of the Universal Protocol (4).

**Table 1:** Causes of wrong site surgery in the OR.

**Causes**
When the same provider performs multiple procedures, there is no intraoperative site verification.
Hand-off communication or briefing process is ineffective.
Primary documentation is not used to verify patient, procedure, site and side immediately prior to incision.
Site marks are removed during prep.
Distractions and rushing occur during time-out, or the time-out occurs before all staff members are ready or before prep and drape.
Time-out is performed without full participation.

## Materials and Methods

The StartBox Patient Safety System (StartBox, Atlanta, GA) is an innovative technology that was evaluated to assess its ability to prevent WSS. This evaluation was performed using cases performed by 11 surgeons at six sites with the StartBox System. The System collects procedure data including a description of the surgery, evaluation of any near misses or other safety benefits added to the case by StartBox, and postoperative evaluation of the occurrence of WSS events defined as any procedure performed at the wrong site including, incorrect procedures, and procedures performed on the wrong patient.

**Figure 1:**
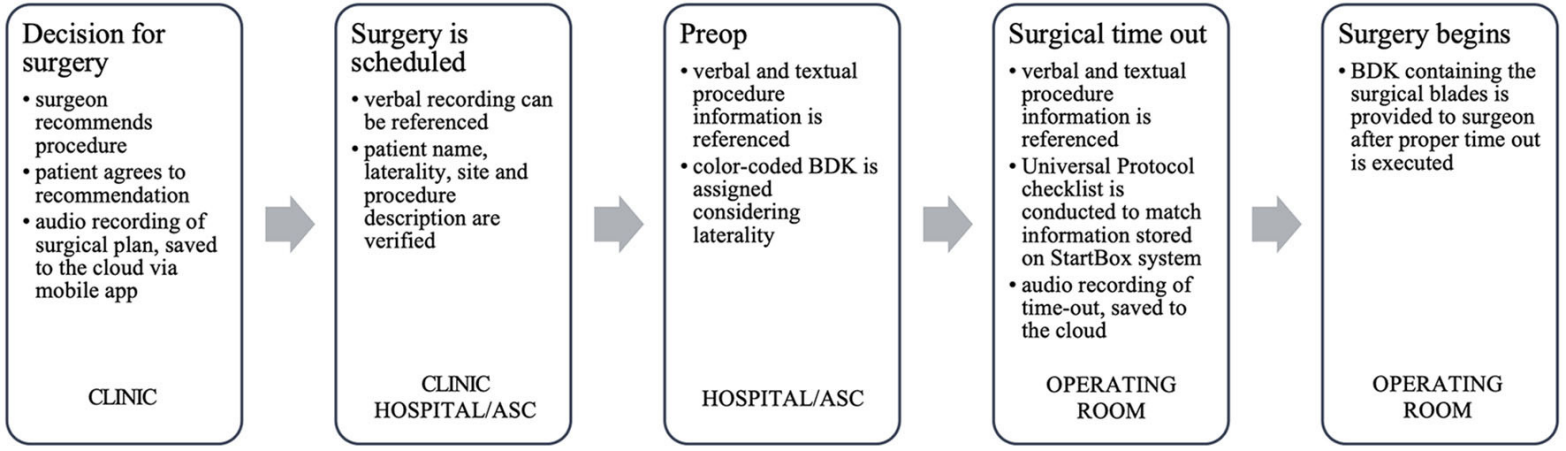
Use of the starBox system along the patient care continuum.

**Figure 2:**
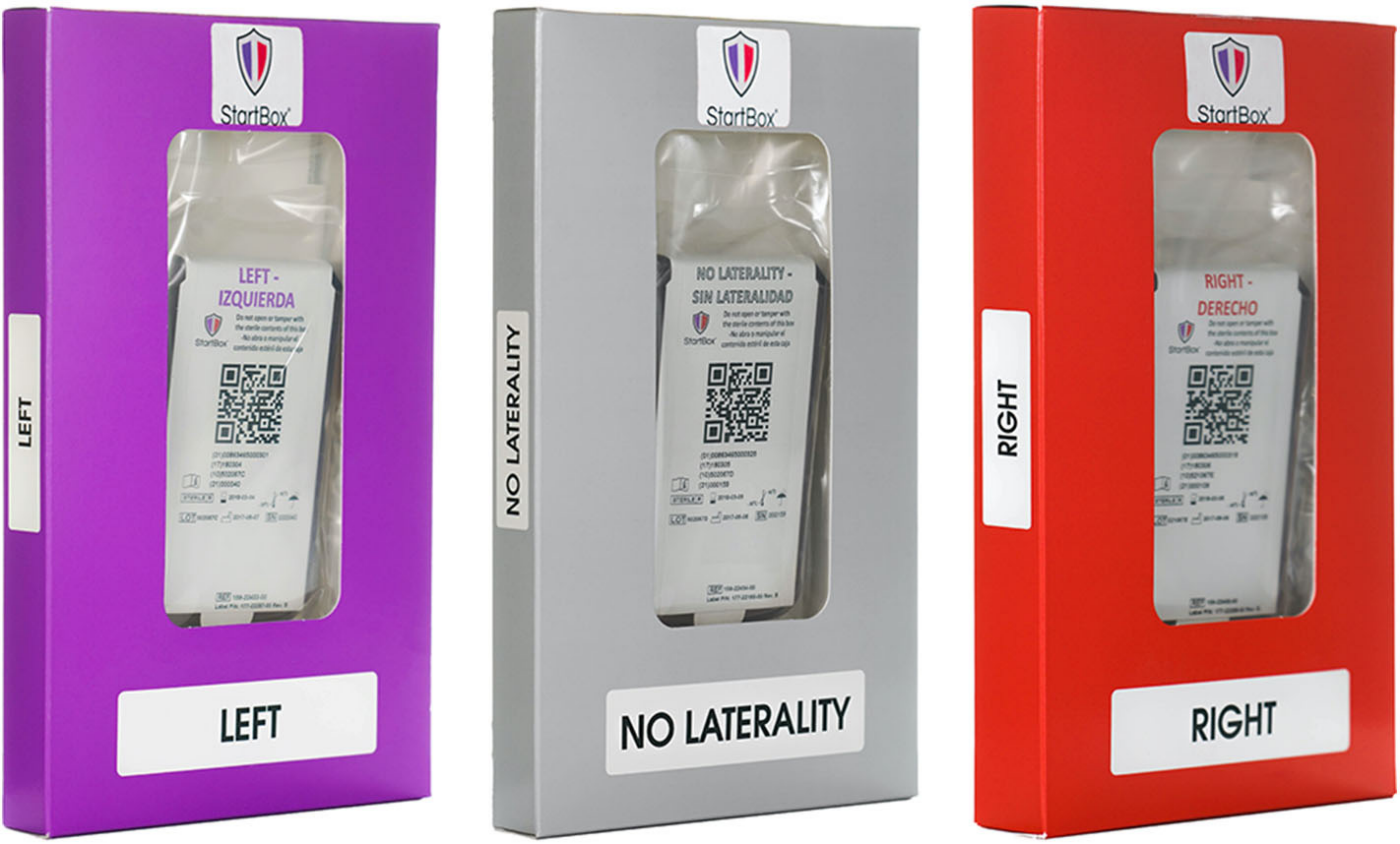
Color-coded startBox blade delivery kits.

**Figure 3:**
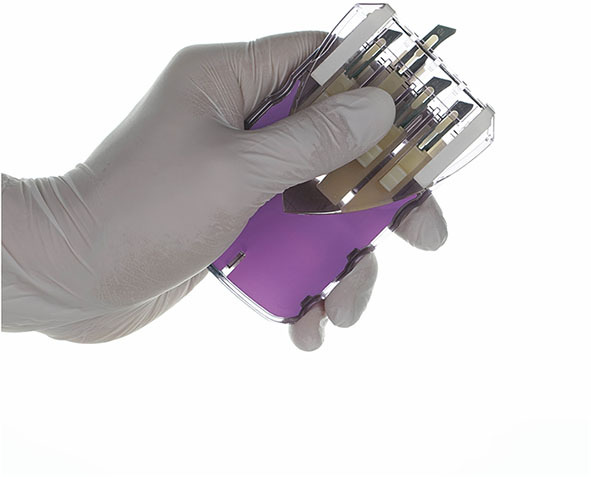
StartBox blade delivery kit ejecting scalpel blades.

The StartBox System consists of a mobile software application, a safety-engineered blade delivery kit (BDK) and a data reporting tool. The software application of StartBox is an easy-to-implement, standardized platform that improves communication between the surgical team and the patient; between the surgeon's practice and surgical facility; and among care providers along the patient care continuum (Figure 1). The application can be loaded on an individual user's personal device, or pre-loaded on a dedicated device provided by the company. The application is compatible with both iOS (Apple) and Android operating systems. The StartBox System is initiated via the mobile application with an audio recording of the surgeon describing the planned procedure to the patient, including site and laterality. The audio file is uploaded to a cloud-based system and becomes accessible to all users of the system, serving as the central source of documentation for the planned surgery. Upon hospital check in, the StartBox patient record is referenced to confirm the correct procedure. Subsequently, the patient's hospital wristband is scanned and associated with a StartBox BDK labeled with a QR code that references the patient's unique procedure, site, and laterality. The packaging of the BDK is color-coded for ready identification of laterality: *Lavender*, for Left; *Rose*, for Right; *Neutral Gray*, for No Laterality (Figure 2). This color-coding is also used in the StartBox software application, which helps prevent the most common type of WSS. The saved audio recording of the decision for surgery is replayed in the preoperative holding area, and in the operating room where surgical personnel listen to the agreed-to procedure discussion just prior to the surgical time-out. At any time between initial consultation in clinic and the start of the procedure in the operation room, StartBox allows for additional voice recordings and playback to remove ambiguity and elaborate on a procedure. Any member of the medical staff can flag errors with the use of a *No Go* function in the StartBox application, which generates a real-time alert in the system, and all *No Gos* must be resolved before the surgery can be initiated. Immediately prior to surgery, the time-out, which includes identification of the patient, site of surgery, and procedure, is conducted as prescribed by the Universal Protocol. This time out is recorded by the application as an additional audio file that is saved to the cloud system to document this confirmation. Upon successful completion of time-out requirements, the BDK is placed on the sterile field. The BDK contains four sterile scalpel blades and delivers each blade in a safe manner, minimizing the potential for sharps injury (Figure 3). With the StartBox System, the BDK serves as a key constraint: the blade for first incision is not delivered to the surgeon until the patient's identity, correct procedure, correct site and correct laterality have been confirmed and documented by the surgical team during the time-out.

Upon completion of the procedure, the case data, including near misses, is stored and aggregated to generate predictive analytics related to future WSS prevention protocol improvements and training opportunities. Near misses related to WSS would include incorrectly booked surgery and improperly performed presurgical time-outs (11).

The study was carried out with sequential series at each site using retrospective, deidentified data. The system is designed to protect the confidentiality, availability, and integrity of personal health information as required by HIPAA and satisfy the compliance requirements of institutional ethics committees.

## Results

The StartBox System was utilized for 487 orthopedic procedures (Table 2). The procedure types include spine, sports medicine, hand, and joint replacement. There were no occurrences of WSS events.

**Table 2:** Count of procedures by type of result.

**Type of result**	**Total**	**Ankle**	**Clavicle**	**Hand**	**Hip**	**Knee**	**Shoulder**	**Spine**
Registered with StartBox	487	2	1	44	8	66	30	336
Near misses aka No Gos (%)	17 (3%)	0	0	0	0	2 (3%)	1 (3%)	14 (4%)
Postponed	1	0	0	0	0	1	0	0
Wrong site surgeries	0	0	0	0	0	0	0	0

Over the course of these procedures, medical staff recorded 17 *No Gos* (Table 3) in the StartBox System. Information for 16 of these cases was either corrected or overridden by the surgeon and successfully completed; one (1) case was postponed to a later date in order to confirm accuracy. The StartBox System was effective in preventing wrong site surgery for each of these near miss events.

**Table 3:** Count of *No Go* by type.

**Type of No Go**	**Total**	**Percentage of total (%)**
Patient	6	35
Description	4	24
Site	2	12
Laterality	5	29
Total	17	

Six (6) *No Gos* were due to inconsistent patient information including incorrect date of birth information, naming errors, and an incorrectly recorded sex. Six (6) *No Gos* were due to incorrect procedure information including site or description. Five (5) *No Gos* were due to laterality mismatch.

The majority of *No Gos* were recorded by the preop nurse at check-in on the day of surgery (9), followed by the circulating nurse in the OR (4) and the clinic scheduler (3). A surgeon recorded one *No Go* (Table 4).

**Table 4:** Count of *No Gos* by reporting individual or area.

**Individual or area**	**Total**	**Percentage of total (%)**
Hospital preop	9	53
OR circulator	4	24
Clinic scheduler	3	18
Surgeon	1	6
Total	17	

The following examples of *No Gos* are provided for illustration purposes. The First is a revision spine procedure where hardware was to be removed from the left side. The surgeon recorded this in the audio dictation, but the procedure record was saved with no laterality and it was scheduled similarly with no laterality. The preop nurse noted this inconsistency and registered a *No Go* in the StartBox System. Before the patient was prepared for surgery, the surgeon corrected the record to reflect the left-side approach and a matching *Lavender* StartBox BDK was paired. The surgical time-out was then completed correctly, and the procedure was conducted successfully.

A second example is a total knee arthroplasty intended for the right side. The procedure was later scheduled for the left side and was incorrectly approved by the patient via signed informed consent. On the day of surgery, the *No Go* was registered with the StartBox System and the team was notified. Due to the laterality discrepancy, the procedure was canceled. Subsequently, the right side was confirmed as the correct operative site.

Two final examples of *No Gos* were spine cases containing incorrect procedure information. The first had improperly transcribed levels. The inconsistency was flagged at preop (C7 was omitted in a C5, C6, and C7 posterior cervical fusion). The second was a spine case with a mismatched informed consent and dictated preoperative surgical plan (the informed consent described a discectomy and the preoperative surgical dictation described a fusion). The patient identified the error after listening to the dictation at the hospital before being prepped for surgery and inquired with the nurse, who then confirmed with the surgeon. The informed consent was then corrected at preop. Both of these procedures were successfully completed with the StartBox System.

Use of StartBox did not result in any reported impacts or use impedance to patient workflow at the clinic or hospital and there were no delays in surgery due to technical difficulties during the time-out or failure of the system to release a surgical blade after completion of the time-out.

## Discussion

### Preventing Wrong Site Surgery

British psychologist James Reason suggested in his Swiss Cheese model of accident causation that catastrophic safety failures are almost never caused by isolated errors committed by individuals. Instead, most accidents result from multiple, smaller errors in environments (the holes in the cheese) with serious underlying system flaws. In this model, errors made by individuals result in disastrous consequences. Reason also emphasized that human error is inevitable, and that a systems approach can catch errors before they occur or block them from causing harm (12).

The Hierarchy of Intervention Effectiveness, first introduced in 1999 by the Institute for Safe Medication Practices, presents a risk management theory that ranks intervention methods from least to most effective. Human-focused interventions such as education, training, rules, policies, and checklists are rated toward the bottom of its scale (13). While not without value, these interventions are less reliable than system-focused interventions such as standardization and computerization. The most highly ranked intervention measures are forcing functions and constraints as they directly prevent the user from making a mistake, thus making them the most powerful and effective error prevention tools (14). Classic examples of forcing functions include a user being prevented from starting a car while it is in gear; or a user being prevented from starting a microwave with the door open (15).

Considering this background, current measures fall short in two categories. First, WSS prevention protocols do not address potential sources of error, according to the Swiss Cheese model. Moreover, relying on a checklist without computerization or constraints is an inferior and generally acknowledged less-effective means of error prevention.

With the end goal of improving WSS prevention protocols and ideally eliminating WSS altogether, the StartBox System was developed to enhance all three intervention methods. System-focused improvements include standardizing and streamlining workflows, as well as complementing existing electronic medical record systems. This system boosts human-oriented methods that contribute to effective communication, such as the huddle described in TeamSTEPPS (Team Strategies and Tools to Enhance Performance and Patient Safety) (16) as well as integrate the checklist recommended by the Universal Protocol (17). Finally, and most importantly, this system adds a physical forcing function as a final constraint prior to the point of no return (the surgical incision).

The goal of this study is to report the early experience using this innovative system comprising the recording of the decision for surgery, verification of the procedure record for all the constituents in the patient care continuum, confirmation of procedure accuracy during the surgery time out, and the use of a physical forcing function before the surgical incision.

In the clinical evaluation that is the focus of this report, StartBox was utilized with 487 procedures to standardize a process that is intended to prevent WSS. Users reported a good experience using the system, including the anecdotal feedback summarized in Table 5. Zero wrong site surgeries occurred.

**Table 5:** Summary of feedback from practitioners.

**Category**	**Comment(s)**
Patient engagement	Patients love hearing surgeon voice on day of surgery (during playback of recording in preop). Gives confidence to them that best care is being provided, and that safety is paramount.
Learning curve	There is a nominal learning curve to using the technology, like anything new. Once overcome it is easy to use and inobtrusive to staff.
Increased efficiency	Does not add material time to clinic phase or hospital. Ensures proper surgical order is placed early in process, minimizing future corrections. Any clarification needed at hospital can be made in preop, before patient goes to operating room. In the OR, the staff realized it made the timeout(s) more efficient.
Staff engagement	Leveling the hierarchy; everyone is in charge of safety. No secrets in patient care; everyone gets to hear the intended procedure.

### Capturing Near Misses

Near misses, sometimes referred to as close calls or potential adverse events, are defined as acts of commission or omission that could have harmed the patient but did not cause harm as a result of chance, prevention or mitigation (18). Near miss analysis is the review of types and causes of error and an investigation of how those errors were mitigated. This type of analysis can contribute toward preventing never events, such as WSS (19).

As a surrogate for WSS, analysis of near miss events may allow organizations to examine the effectiveness of complex systems designed to prevent WSS, without such an event ever occurring (9). A system to capture and analyze near miss data would present a substantial opportunity to reduce or eliminate WSS.

The *No Go* function of the StartBox System was triggered in 17 of 487 (3%) registered procedures. Quantifying near misses is an important step in understanding the risk of WSS and facilitating a longitudinal review of modified systems and protocols to prevent errors. These errors could have been mitigated by standard prevention protocols, but likely not tracked or reported. Near miss analysis is rare (11). There is limited data on the frequency of near misses, which challenges the ability of institutions to examine existing safety systems. The StartBox System inherently captures this data and allows for near miss analysis. The data generated by the StartBox System may improve the safety of future procedures by identifying opportunities for improvement in communication, workflow, logistics, and training. It also provides a unique complement, rather than competitor, to current safety guidelines and protocols employed at any institution.

For example, in this study there were inconsistencies reported between how the procedure was defined by the surgeon during consultation with the patient preop, and how the hospital described it in its OR schedule. The StartBox System highlighted the differences and ensured a resolution through a change to the procedure description used by the hospital. Of particular concern for spine procedures is wrong-level surgeries. The StartBox System did highlight one near miss of this type, ensuring the correct level was performed. Furthermore, spinal procedures could be considered to have no laterality since they are performed generally on the midline of the body; however, there is frequent laterality to the pathology (e.g., left-sided disc herniation), requiring different room setup or approach to the spine after a midline incision. The StartBox System allowed for further precision to be communicated to the OR team by the surgeon, which was an improvement over previous processes. The system color-coding especially supported the awareness of procedure laterality (including room setup and approach) and contributed to the prevention of this frequent type of WSS.

### Study Limitations

The number of patients in the study is small relative to the volume of surgical procedures at a given institution. The small number of patients limit an effective comparison between sites as well as perform any correlation analyses. Future studies with larger number of patients should provide better opportunity to do so. Additionally, the study at each site was conducted over a relatively short period of time, limiting the ability of the institution to perform in-depth analysis of the near misses and implement considerable systematic change or evaluate effectiveness of changes. Continued evaluation with an increasing number of patients over a longer period of time should help further the appreciation for the incidence of near misses and validate the StartBox System as a robust safety system for WSS prevention.

## Conclusion

StartBox is designed to prevent wrong site surgery and capture near misses through a real-time, data-driven approach. The system is designed to complement safety checklists, standardize and streamline workflows, integrate computerization, and provide a final constraint to prevent WSS.

This evaluation included 487 surgical procedures during which StartBox successfully performed all tasks without technical errors and identified 17 near miss events that could have led to the occurrence of a wrong site surgery. Zero wrong site surgeries occurred in this study.

## Data Availability Statement

The raw data supporting the conclusions of this article will be made available by the authors, without undue reservation.

## Ethics Statement

Ethical review and approval was not required for the study on human participants in accordance with the local legislation and institutional requirements. Written informed consent for participation was not required for this study in accordance with the national legislation and the institutional requirements.

## Author's Note

The opinions or assertions contained herein are the private views of the authors and are not to be construed as official or reflecting the views of the Department of Defense or the US government. DG is an employee of the US government. This work was prepared as part of their official duties and as such there is no copyright to be transferred.

## Author Contributions

DG, BH, and DGS performed procedures that are its subject and wrote sections of the manuscript. All authors contributed conception and design of the research, contributed to manuscript revision, read, and approved the submitted version.

## Conflict of Interest

BH, DGS, and JD have equity investment in StartBox, LLC. DS is a consultant for StartBox, LLC. The remaining author declares that the research was conducted in the absence of any commercial or financial relationships that could be construed as a potential conflict of interest.

## References

[1] Devine J Chutkan N Norvell DC Dettori JR. Avoiding wrong site surgery: a systematic review. Spine. (2010) 35(Suppl. 9):S28–36. 10.1097/BRS.0b013e3181d833ac 20407349

[2] Devine J Chutkan N Norvell DC Gloystein D. An update on wrong-site spine surgery. Global Spine J. (2020) 10(Suppl. 1):41S−4. 10.1177/2192568219846911 31934519 PMC6947675

[3] Hall MJ Schwartzman A Zhang J Liu X. Ambulatory surgery data from hospitals and ambulatory surgery centers: United States, 2010. Natl Health Stat Rep. (2017) (Suppl. 102) 102:1–5. 28256998

[4] James MA Seiler JG III Harrast JJ Emery SE Hurwitz S. The occurrence of wrong-site surgery self-reported by candidates for certification by the American Board of Orthopaedic Surgery. J Bone Joint Surg Am. (2012) 94(Suppl. 1):e2. 10.2106/JBJS.K.00524 22218388

[5] Seiden SC Barach P. Wrong-side/wrong-site, wrong-procedure, and wrong-patient adverse events: are they preventable? Arch Surg. (2006) 141(Suppl. 9):931–9. 10.1001/archsurg.141.9.931 16983037

[6] Number of All-Listed Procedures for Discharges From Short-Stay Hospitals by Procedure Category Age: United States. (2010). Available online at: https://www.cdc.gov/nchs/data/nhds/4procedures/2010pro4_numberprocedureage.pdf (accessed November 28, 2017).

[7] Clarke JR Johnston J Finley ED. Getting surgery right. Ann Surg. (2007) 246(Suppl. 3):395–403., discussion 403–5. 10.1097/SLA.0b013e3181469987 17717443 PMC1959354

[8] Canale ST. Wrong-site surgery: a preventable complication. Clin Orthop Relat Res. (2005) (Suppl. 433) 433:26–9. 10.1097/01.blo.0000159827.93813.53 15805933

[9] Agency for Healthcare Research and Quality Patient Safety Network Universal Protocol for Preventing Wrong Site, Wrong Procedure, Wrong Person Surgery. (2003). Available online at: https://www.jointcommission.org/standards_information/up.aspx (accessed April 11, 2019).

[10] Health Research & Educational Trust and Joint Commission Center for Transforming Healthcare Reducing the Risks of Wrong-Site Surgery: Safety Practices From The Joint Commission Center for Transforming Healthcare Project. Chicago, IL: Health Research & Educational Trust (2014). Available online at: www.hpoe.org (accessed December 4, 2017).

[11] Yoon RS Alaia MJ. Using “Near misses” analysis to prevent wrong site surgery. J Healthcare Qual. (2015) 37(Suppl. 2):126–32. 10.1111/jhq.12037 24033453

[12] Agency for Healthcare Research and Quality Systems Approach. (2017). Available online at: https://psnet.ahrq.gov/primers/primer/21/systems-approach (accessed December 6, 2017).

[13] Medication Error Prevention “Toolbox”. (1999). Available online at: https://www.ismp.org/newsletters/acutecare/articles/19990602.asp (accessed December 4, 2017).

[14] Cafazzo JA St-Cyr O. From discovery to design: the evolution of human factors in healthcare. Healthc Q. (2012) 15:24–9. 10.12927/hcq.2012.22845 22874443

[15] Institute of Medicine (US) Committee on Quality of Health Care in America Kohn LT Corrigan JM Donaldson MS, editors. To Err is Human: Building a Safer Health System. Creating Safety Systems in Health Care Organizations. Washington, DC: National Academies Press (2000). p. 8 Available online at: https://www.ncbi.nlm.nih.gov/books/NBK225188/ (accessed December 4, 2017). 25077248

[16] Pocket Guide: TeamSTEPPS – Team Strategies & Tools to Enhance Performance and Patient Safety (2013). Available online at: https://www.ahrq.gov/teamstepps/instructor/essentials/pocketguide.html (accessed May 23, 2019).

[17] The Joint Commission Speak Up. (2018). Available online at: https://www.jointcommission.org/assets/1/18/UP_Poster1.PDF (accessed May 23, 2019).

[18] Bates DW Boyle DL Vander Vliet MB Schneider J Leape L. Relationship between medication errors and adverse drug events. J Gen Intern Med. (1995) 10:199–205. 10.1007/BF02600255 7790981

[19] Aspden P Corrigan JM Wolcott J Erickson SM Patient Safety: Achieving a New Standard for Care. Washington, DC: National Academies Press (2004). p. 11. 25009854

